# Effects of summer treatments against *Varroa destructor* on viral load and colony performance of *Apis mellifera* colonies in Eastern Canada

**DOI:** 10.1093/jisesa/ieae042

**Published:** 2024-05-28

**Authors:** Laurence Plamondon, Marilène Paillard, Carl Julien, Pascal Dubreuil, Pierre Giovenazzo

**Affiliations:** Centre de recherche en sciences animales de Deschambault (CRSAD), Deschambault, Québec, Canada; Centre de recherche en sciences animales de Deschambault (CRSAD), Deschambault, Québec, Canada; Centre de recherche en sciences animales de Deschambault (CRSAD), Deschambault, Québec, Canada; Faculté de médecine vétérinaire, Université de Montréal, St-Hyacinthe, Québec, Canada; Département de Biologie, Université Laval, Québec, Québec, Canada

**Keywords:** formic acid, honey bee, IPM, oxalic acid, varroa, virus

## Abstract

Despite the use of various integrated pest management strategies to control the honey bee mite, *Varroa destructor*, varroosis remains the most important threat to honey bee colony health in many countries. In Canada, ineffective varroa control is linked to high winter colony losses and new treatment options, such as a summer treatment, are greatly needed. In this study, a total of 135 colonies located in 6 apiaries were submitted to one of these 3 varroa treatment strategies: (i) an Apivar® fall treatment followed by an oxalic acid (OA) treatment by dripping method; (ii) same as in (i) with a summer treatment consisting of formic acid (Formic Pro™); and (iii) same as in (i) with a summer treatment consisting of slow-release OA/glycerin pads (total of 27 g of OA/colony). Treatment efficacy and their effects on colony performance, mortality, varroa population, and the abundance of 6 viruses (acute bee paralysis virus [ABPV], black queen cell virus [BQCV], deformed wing virus variant A [DWV-A], deformed wing virus variant B [DWV-B], Israeli acute paralysis virus [IAPV], and Kashmir bee virus [KBV]) were assessed. We show that a strategy with a Formic Pro summer treatment tended to reduce the varroa infestation rate to below the economic fall threshold of 15 daily varroa drop, which reduced colony mortality significantly but did not reduce the prevalence or viral load of the 6 tested viruses at the colony level. A strategy with glycerin/OA pads reduced hive weight gain and the varroa infestation rate, but not below the fall threshold. A high prevalence of DWV-B was measured in all groups, which could be related to colony mortality.

## Introduction

Canadian beekeepers suffer significant winter honey bee (*Apis mellifera* L.) colony losses, ranging between 15% and 45% over the past 20 years (MAPAQ 2021, Ferland et al. 2022). In Europe as in North America, winter mortality increased with the appearance of the parasite, *Varroa destructor* (Mesostigmata: Varroidae) (Anderson and Trueman 2000), in beekeeping operations (Faucon et al. 2002, Le Conte et al. 2010, Neumann and Carreck 2010). Therefore, specialists and beekeepers consider the presence of *V. destructor* as the main cause of honey bee colony mortality around the world (Carreck et al. 2010, Guzmán-Novoa et al. 2010, Le Conte et al. 2010, McMenamin and Genersch 2015, van der Zee et al. 2015, Kulhanek et al. 2017, Canadian Honey Council 2019).

In Canadian temperate climates, the varroa population grows, as the honey bee population increases in the colony and reaches a peak in August when the queen reduces egg laying. These last cycles of brood will become the winter bees that ensure the survival of the colony until the following spring (Döke et al. 2015). Bees from brood infested with varroa show reduced adult weight (Duay et al. 2003), shorter life expectancy (Boecking and Genersch 2008), malformations in developing organs (Garedew et al. 2004), and, by the end of summer, have not fully developed the physiological characteristics of winter bees (Kovac and Crailsheim 1988, Amdam et al. 2004). Furthermore, varroa are known vectors for many deleterious viruses, such as acute bee paralysis virus (ABPV) (Allen et al. 1986), black queen cell virus (BQCV) (Borba et al. 2022), deformed wing virus (DWV) (Highfield et al. 2009, Berthoud et al. 2010, Genersch and Aubert 2010), Israeli acute paralysis virus (IAPV) (Di Prisco et al. 2011), and Kashmir bee virus (KBV) (Shen et al. 2005). Varroa parasitism exposes and affects honey bee internal tissues at critical stages of brood development (Ball and Allen 1988). This weakens their immune system, thus increasing their vulnerability to viral infections and other pathogens (De Jong et al. 1982, Amdam et al. 2004, Yang and Cox-Foster 2005, Traynor et al. 2020). Researchers also found that varroa can replicate certain viruses such as the DWV (de Miranda and Genersch 2010, Gisder et al. 2018).

To avoid the deleterious impacts of varroa on honey bee colonies, effective acaricide treatments must be applied at key moments during the year and should follow an integrated pest management (IPM) plan (Rosenkranz et al. 2010, Jack and Ellis 2021). A successful IPM strategy will reduce infestation rates below recommended thresholds and ensure economic viability of the beekeeping industry. In Quebec province, Canada, the recommended economic thresholds are 2% in summer or a natural varroa drop of 10 varroa per day and 3% in fall or a varroa drop of 15 varroa per day (MAPAQ 2020). An effective IPM strategy includes regular monitoring of varroa populations using natural varroa drop with sticky boards or the alcohol wash method (Dietemann et al. 2013).

In Canada, most beekeepers apply a combination of various chemical treatments in fall (MAPAQ 2019), but only 2 products are presently registered in the presence of honey supers. The first is the Formic Pro (NOD Apiary Products Ltd, Quinte West, ON, Canada), a 42.25% formic acid (FA) polysaccharide gel strip wrapped in eco-paper, which acts as a wick to control the release of the FA vapors. The second is the Hopguard II (BetaTec Hop products, Washington, DC, USA), a liquid miticide derived from hop compounds and delivered in ready-to-use wet insert strips that contain 4 g of hop beta acids per strip. Summer treatments against varroa are not widespread in Canada even though several studies have shown their potential. Martin et al. (2010) state that mites should be controlled in summer, before winter bee brood production begins. van Dooremalen et al. (2012) showed that low infestation rates during winter bee brood production increase winter survival of colonies, and a survey of 323 beekeepers by Beyer et al. (2018) showed that a combination of summer and fall treatments was associated with lower colony losses.

Oxalic acid (OA) is a natural acaricide widely used since the 1980s against varroa. This hydrophilic acid is naturally present in honey (Vilarem et al. 2021). Its acaricidal effect is optimal in the absence of brood (>90%). It has a limited impact on reproducing varroa, as it is not volatile and does not cross the wax operculum of capped brood (Gregorc and Planinc 2002, Rademacher and Harz 2006, Qadir et al. 2021). Interestingly, varroa is unlikely to develop resistance to OA treatment because of its multiple toxic effects (Maggi et al. 2017). It inhibits enzymes of cellular respiration and is neurotoxic (Pernal and Clay 2015). Some studies have tested various methods using OA in the presence of brood (Jack et al. 2020, Berry et al. 2022). An emerging OA application method is the slow-release OA-impregnated glycerin pads (Maggi et al. 2016, Oliver 2018, 2021, Kanelis et al. 2023). Based on its chemical properties and the results of past research, these pads could potentially ensure long term and continuous release of OA within the colony, gradually acting on emerging varroa from capped brood without contaminating hive products.

The objective of this study was to measure the impact of adding 2 different mid-summer treatments (slow-release OA/glycerin pads or Formic Pro) within a commonly used varroa treatment strategy in Eastern Canada (fall Apivar followed by OA dripping) (Véto-pharma, Palaiseau, France). We measured the treatment efficacy, the colony performance traits, the abundance of 6 viruses (ABPV, BQCV, DWV-A, DWV-B, IAPV, and KBV), and the presence of glycerin, OA, or FA residues in honey.

## Materials and Methods

### Colony Preparation and Management

**Fig. 1.**
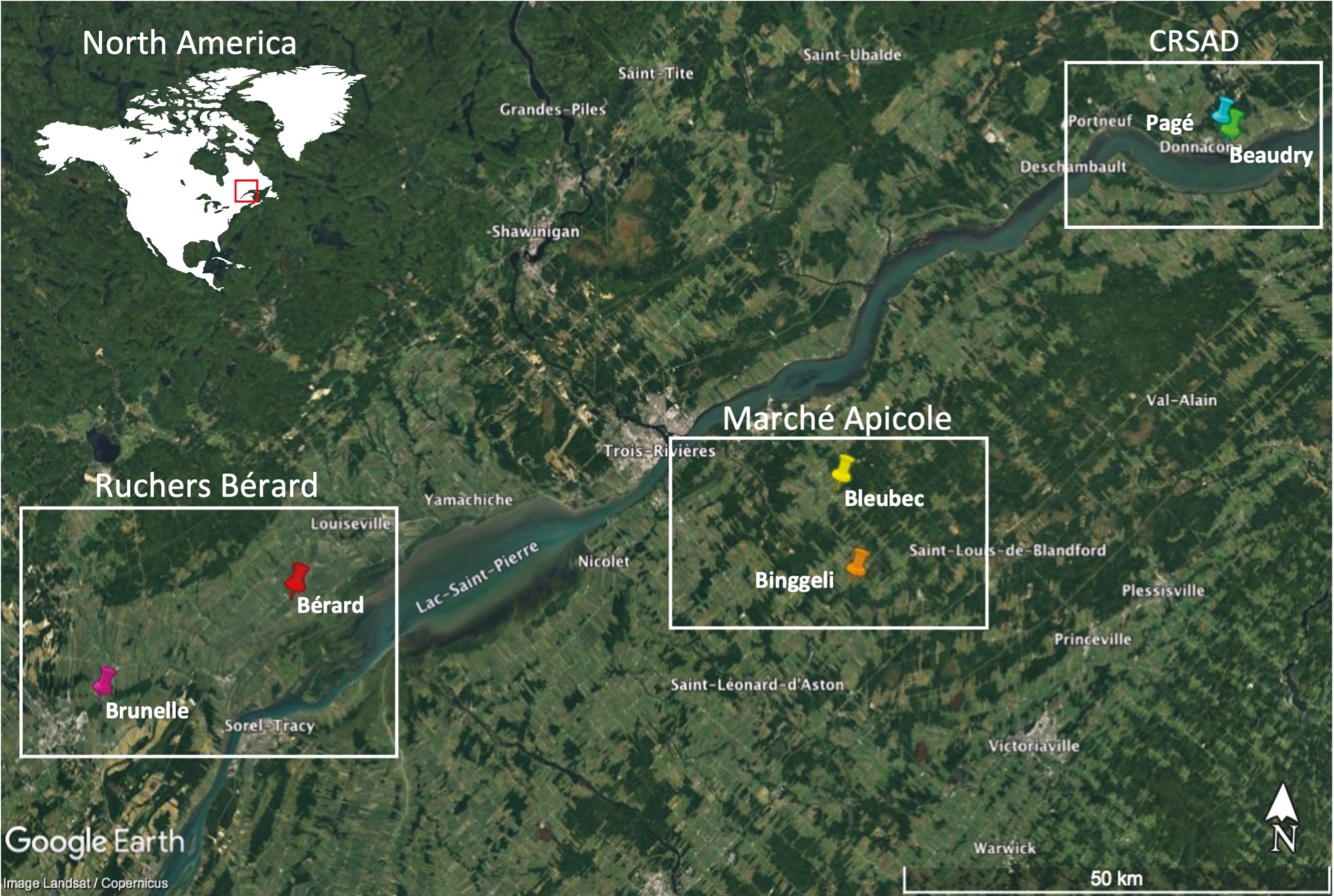
Location of apiaries in the 3 different regions of the province of Quebec (Canada).

Fieldwork was conducted between July 2021 and May 2022. One hundred and thirty-five honey bee colonies were selected among the livestock of 3 beekeeping operations in the province of Quebec (Canada). The Deschambault Animal Sciences Research Center (CRSAD) provided 48 colonies equally distributed in 2 apiaries: Beaudry (46°40ʹ37.9″N 71°42ʹ00.7″W) and Pagé (46°41ʹ22.2″N 71°42ʹ51.0″W); the commercial beekeeper Marché Apicole provided 45 colonies: 24 in the Binggeli apiary (46°12ʹ42.6″N 72°14ʹ38.9″W) and 21 in the Bleubec apiary (46°18ʹ29.9″N 72°16ʹ24.4″W); and the commercial beekeeper Ruchers Bérard provided 42 colonies: 24 were in the Bérard apiary (46°10ʹ17.8″N 73°05ʹ28.8″W) and 18 in the Brunelle apiary (46°03ʹ18.7″N 73 °22ʹ33.5″W) (Fig. 1).

Bee colonies were housed in 10-frame Langstroth hives. All hives were equipped with a varroa screen bottom board (custom-made/Apinovar type). In each apiary, hive entrances were oriented in different directions (at least 10° between consecutive hives) and pieces of geometrically shaped colored plastic were placed on the front panel of hives to minimize the drift of forager bees (von Frisch 1954). Colonies were managed for honey production with a single brood chamber, and honey supers were added over a queen excluder when needed. Colonies were inspected regularly, and queen cells were destroyed to prevent swarming.

### Experimental Protocol

**Fig. 2.**
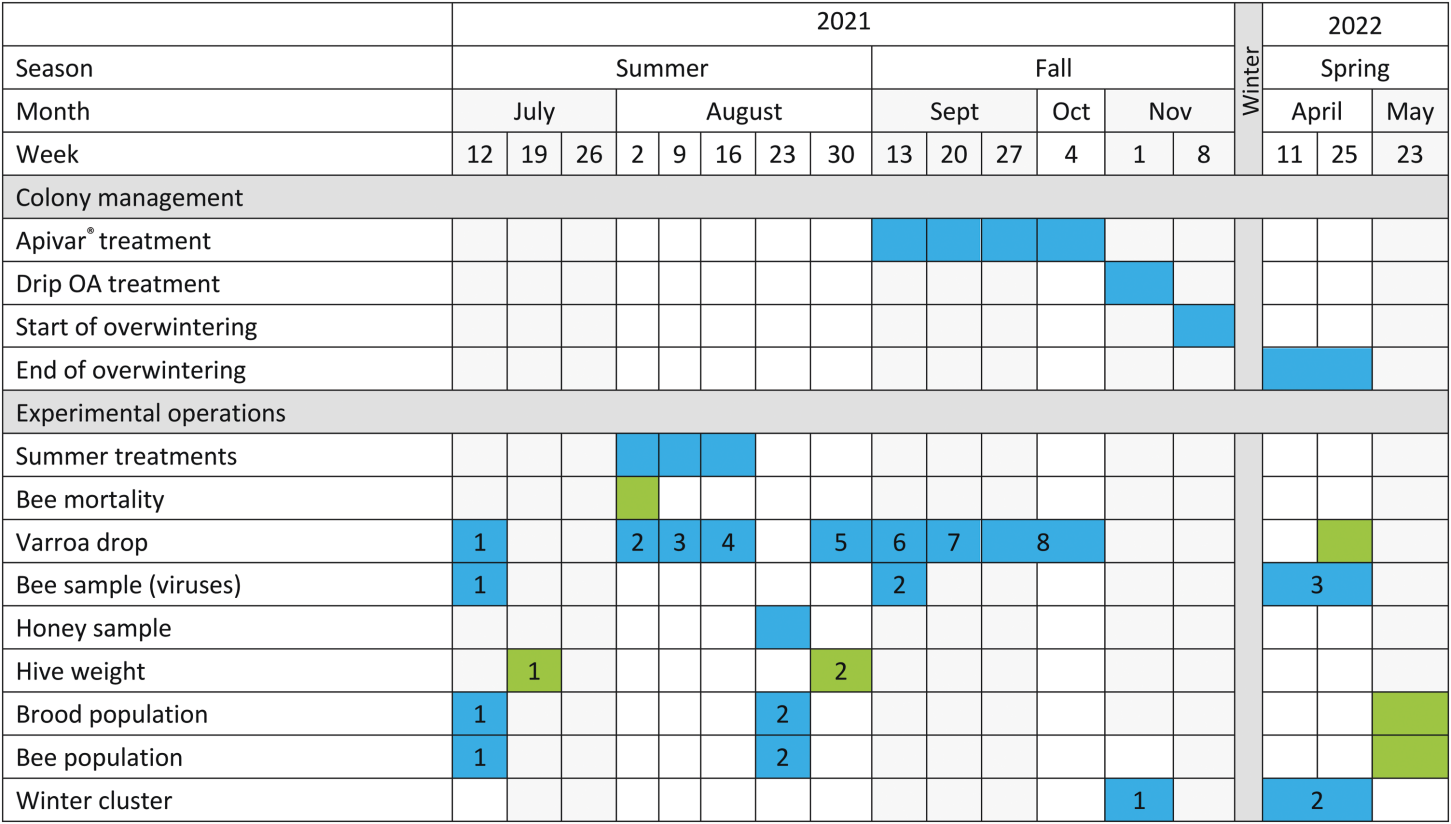
Schedule of manipulations carried out on the colonies in the 3 regions (Deschambault Animal Sciences Research Center [CRSAD], Marché Apicole, and Ruchers Bérard) between July 2021 and May 2022. Boxes indicate when the operations took place, in every regions in blue and in one region (CRSAD) in green, and the numbers indicate the time points for repeated-measures analysis. Colony management includes all procedures normally performed by beekeepers. The experimental operations are specific to this project.

The schedule of manipulations is presented in Fig. 2. On 12 July 2021, the initial varroa infestation rate (daily varroa drop) and the strength (brood and bee population) of each colony were evaluated in order to balance these variables in the 3 experimental groups (Table 1). An equal number of colonies, in their respective apiary, were randomly assigned to one of the 3 experimental groups to be spatially interspersed. At the same time, a first sample of 100 adult worker bees was taken from each colony for viral analyses. On 19 July 2021, CRSAD colonies were weighed.

**Table 1.** Brief description of the different experimental groups

Group	*n*	Varroa treatment strategy		
		Summer	Fall	
Control	45	No summer treatment	Apivar	OA by dripping method
FA	45	Formic Pro		
OA	45	OA/glycerin pads		

**Fig. 3.**
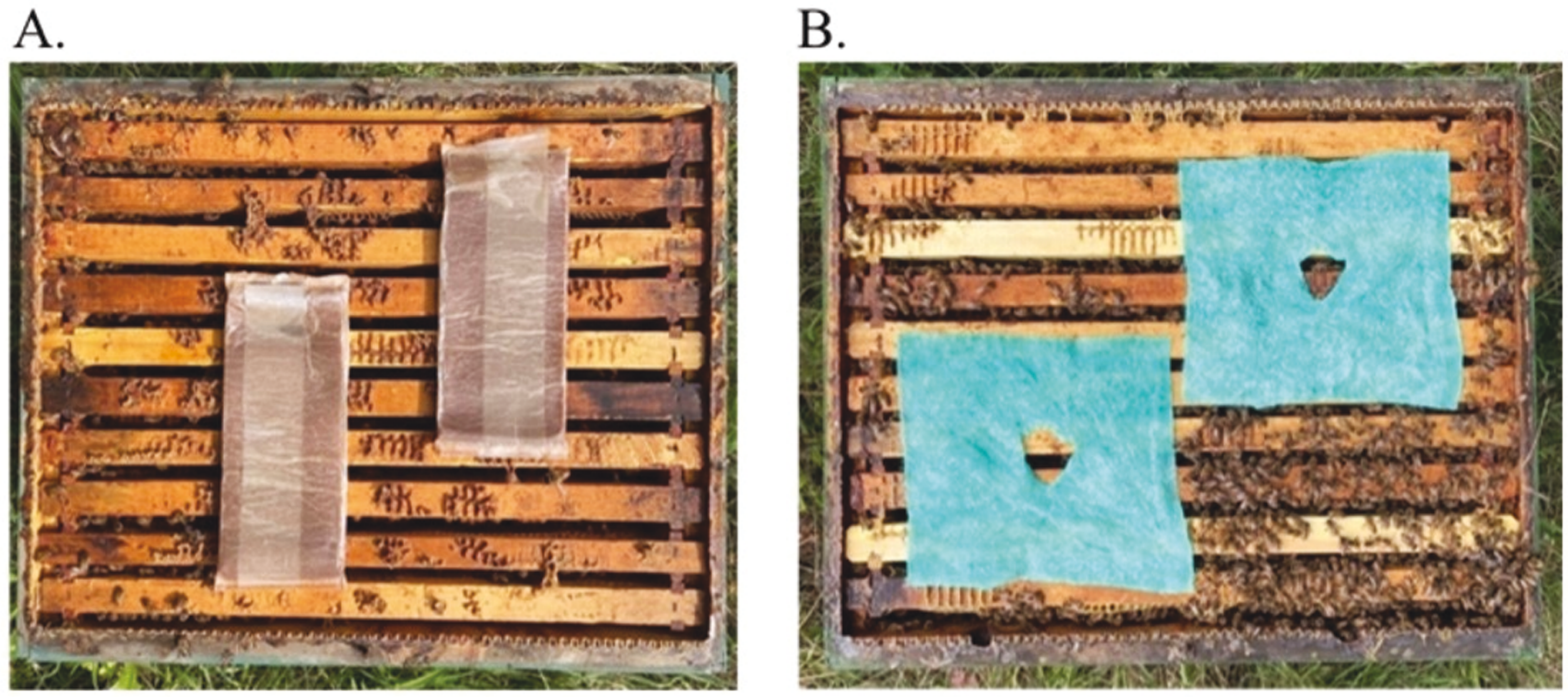
Photographs showing the application of the 2 summer treatments: A) Formic Pro; B) slow-release OA/glycerin pads.

The composition of each group was as follows:

Control group: Colonies without summer varroa treatment but with a fall varroa treatment strategy consisting of an Apivar (Véto-pharma) treatment carried out as per label for 42 days on 13 September 2021, and an OA treatment applied using the dripping method on 1 November 2021: a sucrose solution 50% w/w added with 3.5% OA dihydrate w/v is trickled (5 ml per comb occupied by bees) onto bees between each pair of combs in brood chamber.
FA group: Colonies with the same fall varroa treatment strategy as the control group but with a varroa summer treatment on 2 August 2021, consisting of Formic Pro (NOD Apiary Products Ltd, Quinte West, Ontario, Canada). Treatment was left in place for 21 days. It consists of 2 strips of 10 × 25 cm with 42.25% FA. The strips were arranged diagonally on top of the brood chamber under the queen excluder (Fig. 3A).
OA group: Colonies with the same fall varroa treatment strategy as the control group but with a varroa summer treatment on 2 August 2021, consisting of slow-release OA/glycerin pads. Treatment was left in place for 21 days (Oliver 2018). To make these homemade pads, an OA mother glycerin solution was first prepared by mixing 1 liter of H_2_O and 1 liter of glycerin (99.5% food grade glycerin; Simco Chemicals Inc., Brossard, QC, Canada) on a hot plate, at 60 °C and equipped with a stirrer, until complete dissolution (± 75 min) and then mixed with 1 kg of OA (Univar Canada Ltd, Richmond, BC, Canada, #045420, lot: YP120161017C). The final volume and OA concentration of the mother solution was 2.7 liter and 0.370 g/ml. Second, pads (Wizcloth, UPC: 772548002500) composed of viscose and polypropylene measuring 0.5 mm × 20 cm × 20 cm were saturated with 36.5 ml of the solution to achieve a final quantity of 13.5 g of OA per pad. Each colony was treated by placing 2 pads (27 g of OA per colony) on top of the brood chamber under the queen excluder. A 2 × 2 cm opening in the center of pads was created to facilitate the movement of bees between the supers (Fig. 3B).

On 2 August 2021, the 2 summer varroa treatments (FA and OA groups) were applied simultaneously in the 6 apiaries. Honey bee mortality was monitored during the following 6 days on CRSAD colonies. Varroa drop in all groups was monitored weekly using sticky boards (Mann Lake Bee & Ag Supply, DC681) for a period of 3 weeks. On 23 August 2021, treatments were removed. At the same time, a sample of 50 g of honey was taken from the first honey super and the strength of the colonies was reassessed (brood and bee population). On 30 August 2021, CRSAD colonies were weighed and the varroa infestation rate (daily varroa drop) was reassessed for all colonies and the honey supers were removed. On 13 September 2021, a second sample of 100 adult worker bees was taken from each colony and then the fall varroa treatment strategy started: all colonies of the 3 groups received an Apivar treatment carried out as per label for 42 days. Varroa drop was continuously measured on all colonies using sticky boards changed weekly for the first 2 weeks, then left in place for an additional 2 weeks without changing (total of 4 weeks). On 1 November 2021, OA was applied using the dripping method. On 8 November 2021, all colonies were wintered indoors in environmentally controlled rooms belonging to each beekeeper at an average temperature of 4 °C ± 1 °C and an uncontrolled relative humidity within the range of 55%–70%.

In spring 2022, the Marché Apicole and the Ruchers Bérard colonies were assessed for survival and winter cluster on 11 April 2022. The CRSAD colonies were assessed for survival and winter cluster on 25 April 2022. At the same time, a third sample of 100 adult worker bees was taken from each surviving colony. On 23 May 2022, brood and bee populations (spring recovery) were assessed on surviving CRSAD colonies.

### Dependent Variable Measured

#### Brood population

The number of immature bee workers (eggs, larvae, and pupae) was assessed visually by measuring the area (width × length) on each of the 2 sides of the 10 frames in the brood chamber. The resulting rectangular area was multiplied by 0.8 to compensate for the elliptical shape of the brood pattern. A factor of 25 worker cells per 6.25 cm^2^ was used to convert surface area into the number of immature worker bees (Delaplane et al. 2013).

#### Bee population

The size of the bee population was determined by estimating the number of frames completely covered with bees on the top of the hive (Büchler et al. 2013).

#### Hive weight

Weighing was accomplished by placing the entire hive (brood chamber and honey supers) on a platform scale (total capacity of 500 kg, minimum weight sensitivity of 0.1 kg). Only measured on CRSAD colonies.

#### Winter cluster

The size of the winter cluster was determined by estimating the total number of frames completely covered with bees when temperature was below 10 °C. The number of frames covered with bees on the top and bottom of the hive was noted, then the average was calculated for each hive (Büchler et al. 2013).

#### Bee mortality

A 60 × 60 cm^2^ piece of geotextile fabric was placed in front of each hive. The dead bees found on this surface were counted and removed every day for a period of 6 days following the application of mid-summer treatments. Only measured on CRSAD colonies.

#### Varroa drop

The varroa drop was determined by counting varroa on sticky boards placed under the screened bottom of all hives. The sticky boards were left in place for a period of 7 or 14 days, and all varroa were counted. The daily varroa drop was then calculated by dividing the total number of varroa by the number of days.

#### Treatment efficacy

Efficacy of the treatments was calculated using the equation described by Dietemann et al. (2013):


$$\begin{array}{l} Ef\;f\;icacy\;\%=\;\frac{\;varroa\;drop\;during\;summer\;treatment}{\begin{array}{l} \left(varroa\;drop\;during\;summer\;treatment\;+varroa\;drop\;during\;Apivar\;treatment\right) \end{array}}\times 100 \end{array}$$

#### FA, OA, and glycerin in honey

Capped honey was taken from the first honey super (closest to the treatment) directly from each colony after summer treatments. Honey samples were pooled for every experimental group in each apiary, so there were 6 pooled samples of honey per treatment. Samples were stored in sealed containers and immediately shipped to QSI Laboratories for analysis (Flughafendamm 9a, 28199, Bremen, Germany).

#### Colony mortality

Colony mortality was recorded throughout the protocol. In this study, a colony was considered nonviable and was removed from the protocol when experiencing a queen mortality, and there was no new queen to replace her or when it had 2 frames of bees or less in April. Any data collected prior to this event were used in the statistical analysis.

#### Viruses

The following viruses were quantified: acute bee paralysis virus (ABPV), black queen cell virus (BQCV), deformed wing virus variant A and B (DWV-A and DWV-B), Israeli acute paralysis virus (IAPV), and Kashmir bee virus (KBV). Honey bee samples consisted of 100 adult worker bees taken from a single frame (with brood when present) in the middle of the brood chamber using 50-ml jars certified sterile, then immediately euthanized by placing them on dry ice. All samples were stored at −80 °C until analysis. The main steps of virus analysis are based on standard methods for virus research in *Apis mellifera* (de Miranda et al. 2013) and are summarized below:

Viral RNA extraction was performed using beads cryogenic grinding. Ten bees were put in a 7-ml stainless steel tube for dry grinding (P000952-LYSK0-A.0, Bertin Instruments, Montigny-le-Bretonneux, France) with 2 × 6.8 mm ceramic beads (P000931-LYSK0-A.0, Bertin Instruments) and were kept in liquid nitrogen to ensure the sample remained frozen until the grinding process. The metal tubes were then put in the Precellys Evolution equipped with Cryolys Evolution (P000062-PEVO0-A.0 & P000671-CLYS2-A.0, Bertin Instruments) filled with crushed dry ice for 10 s, at 8,800 rpm with a cooling temperature of 0 °C. Fifty milligrams of the frozen powder obtained was transferred to a SafeSeal reaction tube of 1.5 ml in which 1 ml of TRIzol Reagent (ThermoFisher, Waltham, MA) was added. The mix was then incubated for 5 min at room temperature. Afterwards, the preparation was centrifuged for 10 min at 12,000 × g at 4 °C. Eight hundred microliters of the supernatant was then transferred to a 2-ml RNase DNase-free tube, to which 800 µl of 100% ethanol was added. RNA was then purified using Direct-zol RNA Miniprep (Zymo, Irvine, CA) according to the manufacturer’s instructions. The material was autoclaved between each extraction.

Nucleic acid quantification with 2 µl per sample was performed by 260-nm absorbance measurement by an Infinite M200 PRO with the NanoQuant Plate (Tecan, Mäennedorf, Switzerland). To ensure the purity of nucleic acids, a 260/280 nm ratio of 2.0 or more was required to proceed to the next step. The nucleic acid concentration was calculated automatically by the i-control software (Tecan). The solution was then diluted to a nucleic acid concentration of 62.5 ng/μl, and 1 µg was reverse transcribed using qScript cDNA Supermix (Quantabio, Beverly, MA) in a 20-µl reaction following the manufacturer’s recommendations and using an AriaMx Real-Time PCR System (Agilent, Santa Clara, CA).

The qPCR primers and probes are provided in Supplementary Table 1. New probes were designed using the PrimerQuest Tool (IDT, Newark, NJ). The qPCR analyses were carried out in multiplexes. For this, the 6 viruses were divided into 2 of 3 groups according to affinity. The interactions between the oligos were analyzed with the OligoAnalyzer Tool (IDT, Newark, NJ), for melting temperature and hairpin, and BLAST (NCBI, Bethesda, MD), for specificity. Primers and probes of a housekeeping gene, β-actin, were added to each sample, as an amplification control. The following multiplexes were selected: Multiplex 1: IAPV, BQCV, KBV, and β-actin; Multiplex 2: ABPV, DWV-A, DWV-B, and β-actin. The 2 multiplexes were simulated on SnapGene software (GSL Biotech, San Diego, CA). Preliminary tests were also carried out on 6 samples from highly infested colonies that died in the fall 2019 and 6 samples from low-infested colonies (Cournoyer et al. 2022). Amplification assays were performed in duplicate with 2 µl of cDNA and carried out in a total volume of 20 µl. The primer concentrations were of 500 nM, and the probe concentrations were of 250 nM. The qPCR mix also contained PerfeCta MultiPlex qPCR ToughMix (QuantaBio, Beverly, MA). The quantification was performed by TaqMan real-time quantitative PCR (qRT-PCR) in a AriaMx Real-Time PCR System. Standard curves were prepared from 10-fold serial dilutions (10^1^–10^8^) of DNA fragments harboring the target/reference amplicons (gBlock, IDT), and copy numbers were reported for each amplification. PCR conditions for the Multiplex 1 were as follows: 1 cycle at 95 °C for 3 min for initial denaturation/enzyme activation followed by 40 cycles at 95 °C for 5 s and 60 °C for 45 s. PCR conditions for the Multiplex 2 were as follows: 1 cycle at 95 °C for 3 min for initial denaturation/enzyme activation followed by 40 cycles at 95 °C for 5 s and 60 °C for 20 s. The PCR amplification data were analyzed using AriaMx Software (Agilent) and exported to an Excel spreadsheet.

### Statistical Analyses

Statistical analyses were performed with R (v.4.2) (R Core Team, Vienna, Austria), and the results were interpreted with a significance level of 0.05. Variations of ANOVA models, estimated with linear mixed models (*nlme::lme* [Pinheiro and Bates 2000]; *lme4::lmer* [Bates et al. 2015]) and generalized linear mixed-effect models (*lme4::glmer* [Bates et al. 2015]), with a binomial family and a logit link, were performed according to the experimental design of each variable. Fixed effects included group and, when applicable, time (for time points of each variable, see Fig. 2) and their interaction. Random effects included region (except when measured in only one region), apiary and colony (in the presence of repeated measures). Global tests for fixed effects were obtained using *emmeans::joint_tests* function (Lenth 2022). When a significant difference was found, pairwise comparisons using adjusted Tukey tests were performed (*emmeans::emmeans* and *emmeans::pairs* functions [Lenth 2022]). The normal distribution and the homogeneity of the variances were validated on model residuals with the Shapiro–Wilk test, histogram, and plot of residuals vs. predicted values. In the presence of heteroscedasticity, heterogeneous variances were modeled according to the problematic factor using the *weights* argument.

Brood population and winter cluster data were transformed using a square transformation, while the number of dead bees, residues in honey, and varroa drop data were transformed using a log transformation to meet the normality assumption. In these cases, *P*-values are from the models with transformations, while means and 95% confidence intervals are from the models with untransformed data. For viral load, ranked values were used (*base::rank* function). For treatment efficacy, the number of successes/total number of trials (as explained in the variables section) was considered as the response. For bee population, brood population, and hive weight that were evaluated pretreatment and posttreatment, we compared the temporal evolution between groups (interaction term), and we present this temporal evolution as the change (∆) in each group. Results were plotted using the *ggplot2* package (Wickham 2016).

## Results

### Colony Performance

**Fig. 4.**
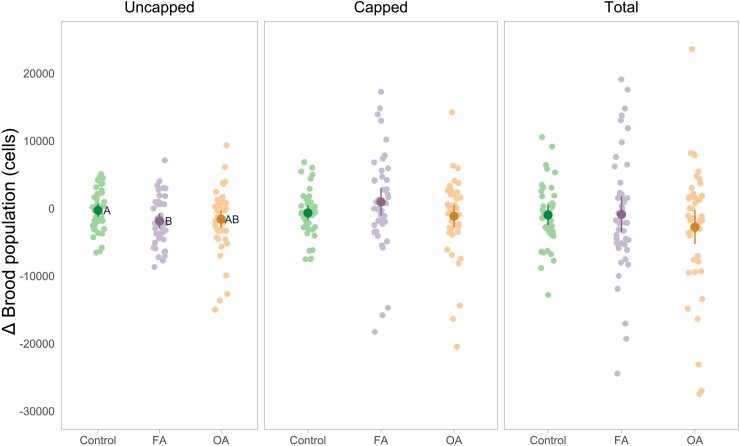
Change (Δ) in honey bee brood population (cells): uncapped (eggs and larvae), capped (pupae), and total in the control group (*n* = 43), FA group (*n* = 45), and OA group (*n* = 45). Measured pretreatment and posttreatment and the Δ calculated for plotting. The mean change and 95% confidence interval were estimated by the model with untransformed data, and each point represents the observed value of a single colony. A statistical difference between groups is indicated by different letters next to the estimate (*P* ≤ 0.05).

For the uncapped brood population evaluated pretreatment and posttreatment, the effect of group (*F*_2,126_ = 4.725, *P* = 0.0105), time (*F*_1,129_ = 13.836, *P* = 0.0003), and their interaction was significant (*F*_2,129_ = 3.134, *P* = 0.0469). The average loss of uncapped brood population of the FA group was significantly greater compared to the control group (mean [95% confidence interval]; −1,850 [−2,939, −762] cells and −274 [−1,235, 688] cells, respectively; *t*_129_ = −2.461, *P* = 0.0400), but was similar to the OA group (−1,594 [−2,863, −324] cells; *t*_129_ = 0.742, *P* = 0.7390). The average loss of the uncapped brood population of the OA group was similar to the control group (*t*_129_ = −1.566, *P* = 0.2639). The temporal evolution of capped and total brood populations was similar between groups (*F*_2,129_ = 1.809, *P* = 0.1680 and *F*_2,129_ = 0.733, *P* = 0.4823, respectively) (Fig. 4). The temporal evolution of the adult honey bee population was also similar between groups (*F*_2,130_ = 0.525, *P* = 0.5925).

**Fig. 5.**
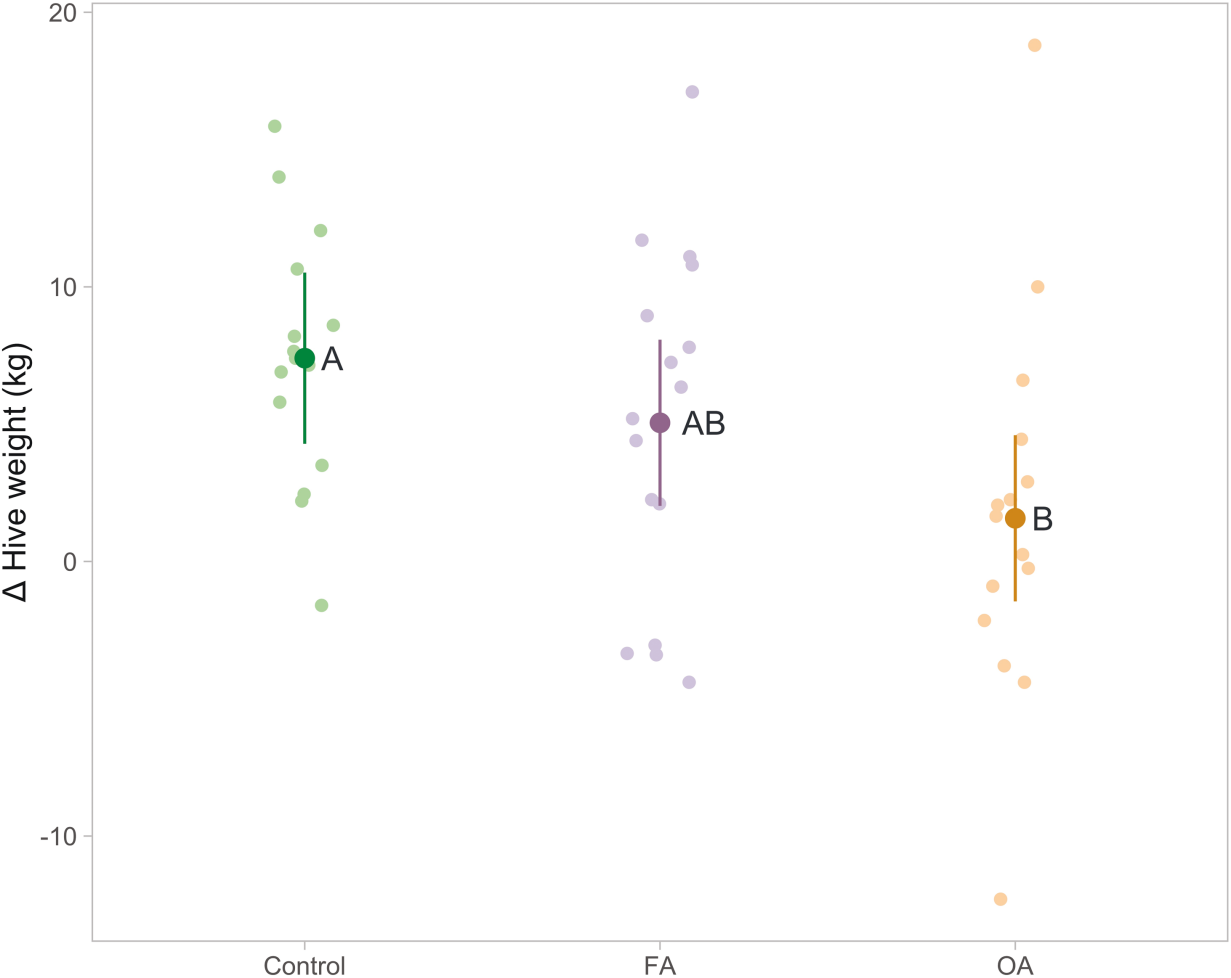
Change (Δ) of hive weight (kilogram) in the control group (*n* = 15), FA group (*n *= 16), and OA group (*n *= 16). Measured pretreatment and posttreatment and the Δ calculated for plotting. The mean change and 95% confidence interval were estimated by the model with untransformed data, and each point represents the observed value of a single colony. A statistical difference between groups is indicated by different letters next to the estimate (*P* ≤ 0.05).

For hive weight, the effect of time (*F*_1,44_ = 28.483, *P *< 0.0001) and the interaction of group and time were significant (*F*_2,44_ = 3.718, *P* = 0.0322), but not the effect of group (*F*_2,44_ = 0.059, *P *= 0.9432). The OA group gained significantly less weight than the control group (1.57 [−1.45, 4.60] kg and 7.40 [4.28, 10.52] kg, respectively; *t*_44_ = −2.704, *P* = 0.0258), but this gain was similar to the FA group (5.05 [2.02, 8.08] kg; *t*_44_ = −1.638, *P* = 0.2409). No difference was found between the FA group and the control group (*t*_44_ = −1.091, *P =* 0.5245) (Fig. 5).

For the winter cluster measured on 1 November 2021 and 11–25 April 2022, the effect of group (*F*_2,100.74_ = 3.213, *P =* 0.0444) and the effect of time (*F*_1,102.93_ = 10.851, *P =* 0.0014) were significant, but not their interaction (*F*_2,100.69_ = 0.088, *P* *=* 0.9159).

For the bee and brood population measured on 23 May 2022 (spring recovery), there was no significant group effect on honey bee population (*F*_2,23.47_ = 1.973, *P* = 0.1614), but the effect of group on brood population was significant (*F*_2,23.26_ = 5.508, *P =* 0.0110). The brood population in FA group was significantly higher than the OA group (mean [95% confidence interval]; 21,202 [10,127, 32,278] cells and 15,191 [6,783, 23,598] cells, respectively; *t*_23.1_ = 3.155, *P* = 0.0118) and was similar to the control group (20,598 [12,677, 28,520] cells; *t*_23.1_ = 0.291, *P* = 0.9546). The brood population in the control group tends to be higher than the OA group, but this difference was not significant (*t*_23.3_ = 2.458, *P* = 0.0549).

### Bee Mortality

For bee mortality in front of the hive, the group effect was significant (*F*_2,43_ = 58.582, *P *< 0.0001). During the 6 initial days of summer treatments, the total number of dead bees collected in front of the hive was significantly higher in the FA group compared to the control group (mean [95% confidence interval]; 357 [249, 465] dead bees and 27 [0, 135] dead bees, respectively; *t*_43_ = 9.439, *P <* 0.0001) and the OA group (27 [0, 135] dead bees; *t*_43.1_ = 9.250, *P <* 0.0001). The total number of dead bees was equivalent for the OA group and the control group (*t*_43.1_ = −0.022, *P =* 0.9997) (Supplementary Fig. 1).

### Varroa

**Fig. 6.**
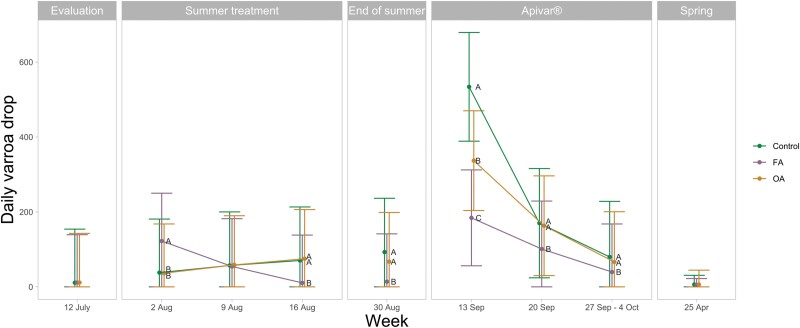
Daily varroa drop measured during 7- or 14-day intervals in the control group, FA group, and OA group from 12 July 2021 until 25 April 2022. The mean and 95% confidence interval were estimated by the model with untransformed data. A statistical difference between groups at each time point is indicated by different letters next to the estimate (*P* ≤ 0.05).

The average daily varroa drop for the various groups at different times is shown in Fig. 6. For varroa drop, the effect of group, time, and their interaction was significant (*F*_2,126_ = 7.758, *P *= 0.0007; *F*_7,884_ = 346.743, *P *< 0.0001; *F*_14,884_ = 33.417, *P *< 0.0001, respectively). During the summer treatment (2 August, 9 August, and 16 August 2021), the daily varroa drop in the FA group during the week of 2 August was significantly higher than the control group (*t*_126_ = 6.034, *P *< 0.0001) and the OA group (*t*_126_ = 5.981, *P* < 0.0001), but the daily varroa drop in the OA group was similar to the control group (*t*_2,126_ = 0.029, *P* = 0.9995). The daily varroa drop in the OA group was also similar to the control group during the week of 9 and 16 August (*t*_126_ = 0.576, *P* = 0.8331; *t*_126_ = 1.204, *P* = 0.4530, respectively). During the week of 16 August, the daily varroa drop in the FA group was significantly lower than the control group and the OA group (*t*_126_ = −7.243, *P* < 0.001; *t*_126_ = −8346, *P* < 0.0001, respectively).

One week after the end of summer treatments (30 August 2021), we measured the daily varroa drop to see whether groups reached the fall IPM threshold of <15 daily varroa drop. The majority of the FA group colonies were brought below the fall IPM threshold of 15 daily varroa drop (33 of 45 colonies), while this was not the case for the control group colonies (7 of 45 colonies) and the OA group colonies (13 of 45 colonies). The daily varroa drop of the FA group was significantly lower than the control group (13.8 [0, 141] daily varroa drop and 92.8 [0, 237] daily varroa drop, respectively; *t*_126_ = −8.313, *P <* 0.0001) and the OA group (67.2 [0,199] daily varroa drop; *t*_126_ = −6.048, *P <* 0.0001). The end-of-summer varroa drop of the OA group tended to be lower than the control group but was not significantly different (*t*_126_ = −2.177, *P =* 0.0791) (Supplementary Fig. 2).

During the Apivar treatment (13 September, 20 September, and 27 September–4 October 2021), daily varroa drop was significantly lower in the FA group at the 3 times compared to the control group (*t*_126_ = −6.618, *P *< 0.0001; *t*_126_ = −4.056, *P *= 0.0003; *t*_126_ = −3.742, *P *= 0.0008; chronologically) and the OA group (*t*_126_ = −4.052, *P *= 0.0003; *t*_126_ = −2.468, *P *= 0.0394; *t*_126_ = −2.456, *P *= 0.0405). The daily varroa drop of the OA group was significantly different from the control group at one of the 3 times (*t*_126_ = −2.443, *P *= 0.0420, *t*_126_ = −1.520, *P *= 0.2851; *t*_126_ = −1.227, *P *= 0.4395).

The following spring (25 April 2022), the daily varroa drop was similar between groups (*F*_2,22_ = 0.205, *P* = 0.8161).

### Summer Treatment Efficacy

**Fig. 7.**
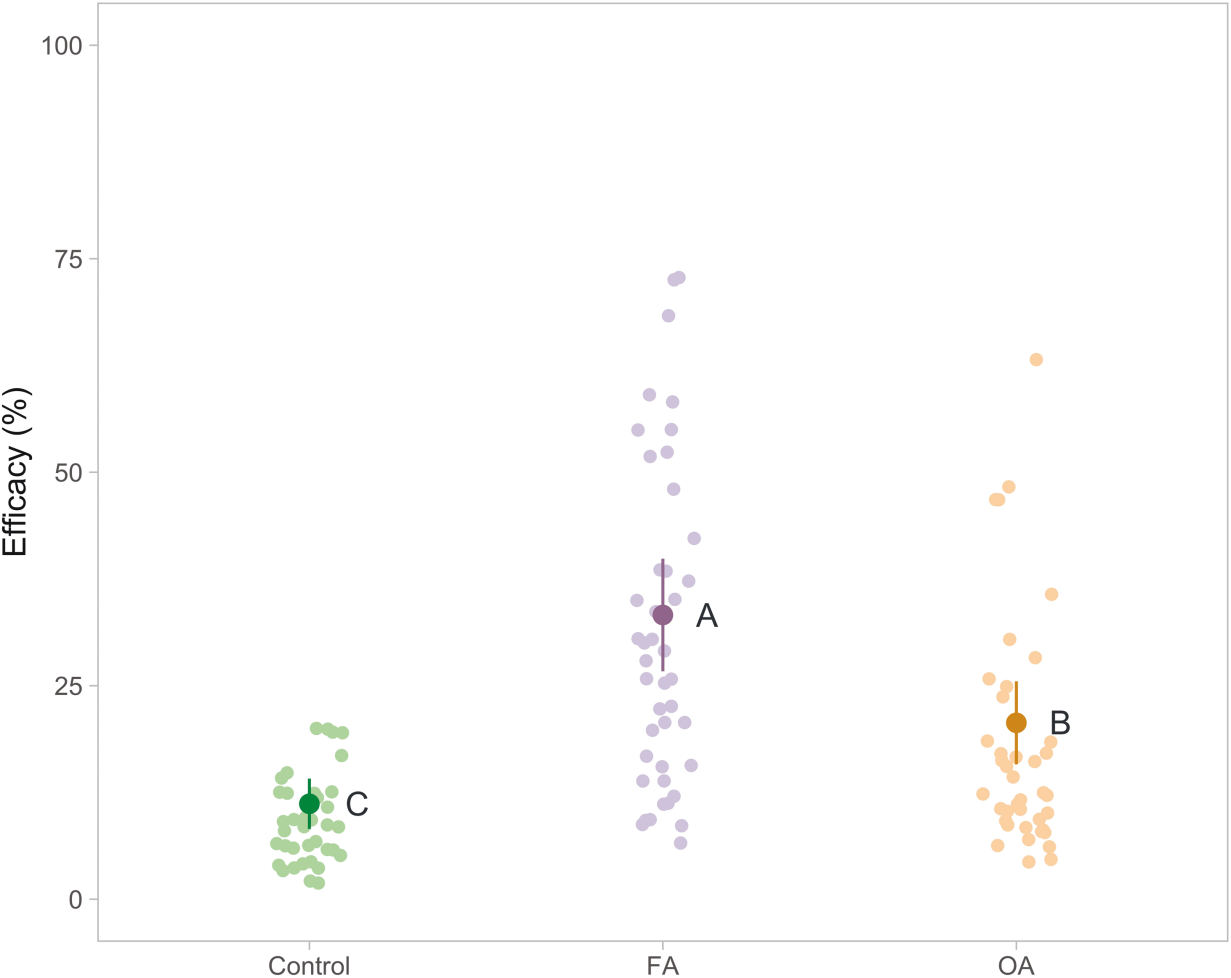
Summer treatment efficacy (%) in the control group (*n* = 37), FA group (*n* = 43), and OA group (*n* = 40). Efficacy = varroa killed during summer treatment/(varroa killed during summer treatment + varroa killed during Apivar treatment) × 100. The mean and 95% confidence interval were estimated by the model with untransformed data, and each point represents the observed value of a single colony. A statistical difference between groups is indicated by different letters next to the estimate (*P* ≤ 0.0001).

For summer treatment efficacy, the group effect was significant (*F*_2,∞_ = 13,600.469, *P* < 0.0001). The efficacy of the 2 experimental summer treatments, FA and OA, was significantly higher than the control group (*z* = 164.032, *P <* 0.0001; *z* ratio = 86.688, *P <* 0.0001; respectively). The efficacy of the FA summer treatment was also significantly higher than the OA summer treatment (*z* ratio = 87.873, *P < *0.0001). The efficacy of the control, OA, and FA summer treatment was, respectively, (mean [95% confidence interval]) 11.2 [8.6, 14.5] %, 20.7 [16.2, 25.9] %, and 33.3 [27.0, 40.2] % (Fig. 7).

### FA, OA, and Glycerin Amount in Honey

**Fig. 8.**
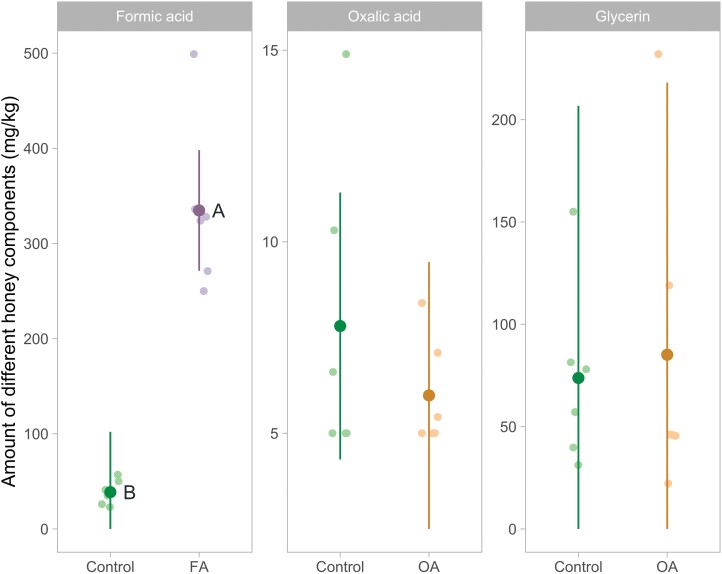
Amount of FA, OA, and glycerin in honey in the control group (*n* = 6), FA group (*n* = 6), and OA group (*n* = 6). Each sample was taken directly from the first honey super of each colony on 23 August 2021. Samples from colonies of the same experimental group were pooled for each apiary. The mean and 95% confidence interval were estimated by the model with untransformed data, and each point represents the observed value of pooled colonies from a single apiary. A statistical difference between groups is indicated by different letters next to the estimate (*P* ≤ 0.05).

For the FA amount in honey, the group effect was significant (*F*_1,5_ = 154.332 *P *= 0.0001). The amount of FA was significantly higher in the FA group compared to the control group (mean [95% confidence interval]; 334.7 [271.3, 398] mg/kg and 38.7 [0, 102] mg/kg, respectively; *t*_5_ = 12.423, *P *= 0.0001). For the OA and glycerin amount in honey, the group effect was not significant (*F*_1,5_ = 1.747, *P =* 0.2435; *F*_1,5_ = 0.012, *P =* 0.9159; respectively) (Fig. 8).

### Viruses

**Fig. 9.**
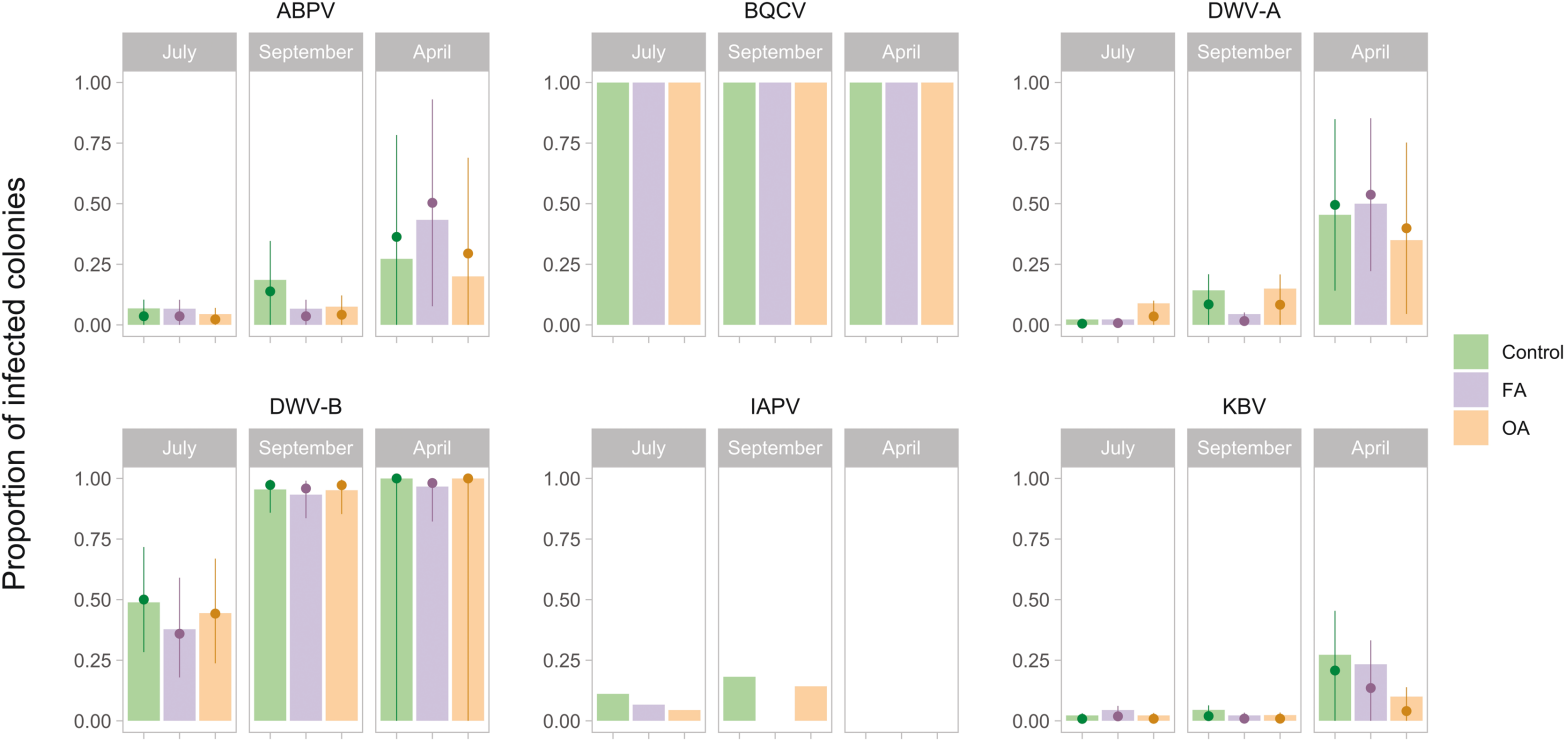
Virus prevalence in colonies at 3 times during the season in the control group (*n* = 45, 44, and 22), FA group (*n* = 45, 45, and 30), and OA group (*n* = 45, 42, and 20). Measured in July 2021, September 2021, and April 2022. The mean and 95% confidence interval were estimated by the model with untransformed data (when possible), and each bar represents the observed value of a single group.

For the prevalence of viruses, few colonies tested positive for IAPV and BQCV were present in all colonies throughout the year; thus, statistical tests for prevalence were not performed for those viruses. For the viruses analyzed, the group effect (ABPV: *F*_2,∞_ = 1.008, *P* = 0.3651; DWV-A: *F*_2,∞_ = 0.945, *P* = 0.3886; DWV-B: *F*_2,∞_ = 1, *P* = 1; KBV: *F*_2,∞_ = 0.526, *P* = 0.5912) and the interaction between time and group (ABPV: *F*_4,∞_ = 1.053, *P* = 0.3783; DWV-A: *F*_4,∞_ = 1.377, *P* = 0.2389; DWV-B: *F*_4,∞_ = 0.007, *P* = 0.9999; KBV: *F*_4,∞_ = 0.378, *P* = 0.8246) were not significant, but for some viruses, the effect of time was significant (ABPV: *F*_2,∞_ = 14.065, *P* < 0.0001; DWV-A: *F*_2,∞_ = 9.642, *P* < 0.0001; DWV-B: *F*_2,∞_ = 27.696, *P* < 0.0001; KBV: *F*_2,∞_ = 7.184, *P* = 0.0007). Not all viruses have the same prevalence dynamics. The prevalence of ABPV and KBV was similar between July and September (*z* = −1.377, *P *= 0.3532; *z* = −0.079, *P* = 0.9966, respectively) and was significantly higher in April than in September (*z* = 4.278, *P* = 0.0001; *z* = 3.176, *P* = 0.0043). The prevalence was also significantly higher in April than in July for those 2 viruses (*z* = 5.064, *P* < 0.0001; *z* = 3.229, *P* = 0.0036). The prevalence of DWV-A tended to be higher in September than in July (*z* = 2.136, *P *= 0.0826) and was significantly higher in April than in September (*z* = 3.884, *P* = 0.0003). The prevalence was significantly higher in April than in July (*z* = 4.233, *P* = 0.0001). The prevalence of DWV-B significantly increased between July and September (*z* = 7.443, *P* < 0.0001) and remained stable between September and April (*z* = 0.004, *P* = 1.0000) (Fig. 9).

**Fig. 10.**
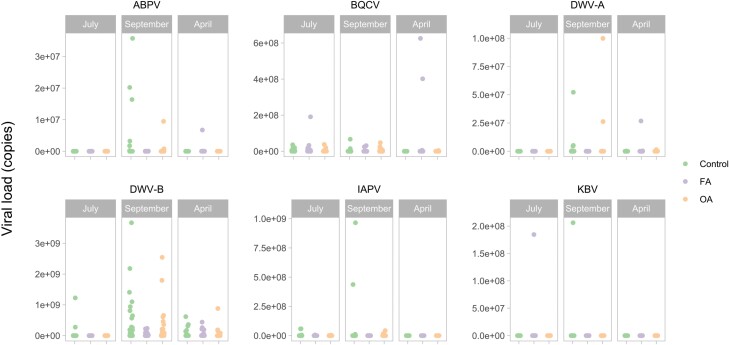
Viral load of colonies (copies) at 3 times during the season in the control group (*n* = 45, 44, and 22), FA group (*n* = 45, 45, and 30), and OA group (*n* = 45, 42, and 20). Measured in July 2021, September 2021, and April 2022. Each point represents the observed value of a single colony.

For the viral load, few colonies tested positive for IAPV and KBV; thus, statistical tests for viral load were not performed for those viruses. For some viruses analyzed, the group effect (ABPV: *F*_2,127_ = 1.154, *P* = 0.3188; DWV-A: *F*_2,127_ = 0.387, *P* = 0.6797) and the interaction between time and group (ABPV: *F*_4,197_ = 2.203, *P* = 0.0701; DWV-A: *F*_4,197_ = 1.870, *P* = 0.1172; DWV-B: *F*_4,197_ = 0.482, *P* = 0.7492) were not significant. For DWV-B, the group effect was significant (DWV-B: *F*_2,127_ = 5.281, *P* = 0.0063), and for BQCV, the interaction between time and group was significant (*F*_4,197_ = 2.990, *P* = 0.0200). The viral load of FA group was significantly higher than the control group in April for BQCV (*t*_2,127_ = 2.833, *P* = 0.0147). For some viruses, the effect of time was significant (ABPV: *F*_2,197_ = 14.065, *P* < 0.0001; BQCV: *F*_2,197_ = 89.273, *P* < 0.0001; DWV-A: *F*_2,197_ = 60.715, *P* < 0.0001; DWV-B: *F*_2,197_ = 60.715, *P* < 0.0001). The viral load for ABPV was similar in September and July (*t*_197_ = −1.687, *P* = 0.2128) and significantly higher in April than in September and July (*t*_197_ = 4.429, *P* < 0.0001; *t*_197_ = 5.834, *P* < 0.0001). The viral load for BQCV was significantly higher in July compared to September and April (*t*_197_ = 12.617, *P* < 0.0001; *t*_197_ = 9.100, *P* < 0.0001, respectively) and was similar between September and April (*t*_197_ = 1.208, *P* = 0.4498). The viral load for DWV-A tended to be higher in September compared to July (*t*_197_ = 2.350, *P* = 0.0515) and was significantly higher in April compared to September and July (*t*_197_ = 5.870, *P* < 0.0001; *t*_197_ = 7.800, *P* < 0.0001). The viral load of DWV-B was significantly higher in September compared to July (*t*_197_ = 9.637, *P* < 0.0001), similar between September and April (*t*_197_ = 0.868, *P* = 0.6614), and significantly higher in April than in July (*t*_197_ = 8.784, *P* < 0.0001) (Fig. 10).

### Colony Mortality

For colony mortality, the group effect was significant (*F* ratio_2,∞_ = 3.619, *P* = 0.0268). Throughout the duration of the study, the probability of mortality was 64.6 [45.4, 79.9] % for the control group, 33.5 [18.6, 52.7] % for the FA group, and 53.5 [34.1, 71.9] % for the OA group. Overall, the FA group had significantly less mortality than the control group (*z* = −2.668, *P* = 0.0208) and similar mortality as the OA group (*z* = −1.690, *P* = 0.2092). The mortality of the OA group and the control group was similar (*z* = 0.968, *P* = 0.5973). All colonies survived during summer (from 12 July to 30 August). In fall (from 30 August to 2 November), 15.9% of the colonies died in the control group, 6.7% in the FA group, and 8.9% in the OA group. During winter (from 1 November 2021 to 25 April 2022), 55.9% of the colonies died in the control group, 28.9% in the FA group, and 43.8% in the OA group.

## Discussion

The varroa treatment strategy with an FA summer treatment (Formic Pro) was the most effective and reduced the varroa drop of the majority of the colonies to below the economic fall threshold of 15 daily varroa drop, which significantly reduced mortality of colonies. The varroa treatment strategy with an OA treatment in glycerin pads was less effective than the FA approach. Further research will be needed to see whether it is possible to increase the effectiveness of this type of treatment. Increasing the concentration of OA and using different materials allowing better contact of the OA with bees and varroa parasites are interesting avenues (Kanelis et al. 2023). An application at the entrance of the hive paired with an application on the top of the brood chamber could also be considered.

The company NOD Apiary Products Ltd, which markets this product in Canada, mentions that Formic Pro has an efficacy between 83% and 97% (NOD Apiary Products 2022). This level of effectiveness was not obtained during this study. The low efficacy we measured (mean [95% confidence interval]); Control: 11.2 [8.6, 14.5] %, OA: 20.7 [16.2, 25.9] %, and FA: 33.3 [27.0, 40.2] %) can be partially explained by the 3-week delay between the end of the summer treatment and the Apivar contrast treatment. During this period, varroa mites that were not killed by the experimental treatment probably continued to reproduce and their population increased. A reinfestation by untreated colonies is also possible during this period. This can occur when a heavily infested colony collapses and bees drift to other colonies or when bees steal resources from collapsing colonies (Peck and Seeley 2019). This could have contributed to underestimate the treatment efficacy, which has also been outlined by Dietemann et al. (2013). To estimate the efficacy of a treatment as accurately as possible, the contrast treatment (the Apivar treatment in this study) must be carried out immediately after the experimental treatment. In the case of this experiment, it was impossible to carry out the Apivar treatment immediately after the experimental treatment for 2 reasons: first, the natural varroa drop at the end of the summer had to be estimated, and second, carrying out a contrast treatment in summer would have interfered with the objective of the experiment, which was to test the efficacy of adding a summer varroa treatment.

### Effects of Summer Treatments on Colonies

In this project, the FA summer treatment significantly affected uncapped brood, but this did not have a significant effect on total brood population. Based on this result, FA treatment seems to have affected queen egg laying and brood rearing for at least 14 days, and this impact could be seen even longer in some colonies since some had no capped brood after 3 weeks. It is well known that FA has negative effects on bees and brood (Tihelka 2018). The OA/glycerin pads summer treatment did not affect the brood significantly. However, we noticed that some colonies had no brood 2 weeks after the treatment. An effect on the queen and/or the brood cannot be ruled out. Although the application of OA in sugar solution has previously been associated with queen mortality (Higes et al. 1999), few studies have been conducted on the toxicity of OA in glycerin pads. In this study, of the 48 queens marked, a single change of queen was observed in the OA group 6 weeks after application. We do not suspect the OA summer treatment to be the cause.

The following spring, on 23 May 2022, there was no significant difference with the control group for both summer treatment groups on colony performance.

The OA/glycerin pads summer treatment significantly reduced hive weight gain, which can be interpreted as reduced honey harvest. OA pads covered more than two-thirds of the brood chamber and could have acted as a barrier impairing the movement of bees between the supers and therefore from going to store honey. Considering that the bees bring back the same quantity of nectar to the hive independently of the space available and the quantity of honey already stored (Fewell and Winston 1996), the honey would then be mainly stored in the brood box. This lower hive weight gain could also be due to OA. Indeed, Evans et al. (2022) also noted a negative effect on the honey yield of colonies that received an OA sublimation treatment that did not include pads.

The FA treatment induced bee mortality even when respecting the recommended outdoor temperature limit. The outdoor temperature obtained by weather stations near the apiaries did not exceed the maximum temperature of the treatment guidelines, which is 29.5 °C. This consequence does not seem to be avoidable in a Canadian climate. The southernmost regions of Canada are more likely to exceed the maximum recommended temperature for this summer treatment, and this could limit its use. An effective treatment without maximum temperature constraints, such as an OA treatment, would be ideal for these regions.

No bee mortality was observed in front of hives treated with OA. Although it has been shown that the application of OA in sucrose solution causes worker and colony mortality (Tihelka 2018), we did not observe any mortality of this kind in our study. Therefore, the application of OA in slow-release OA/glycerin pads appears to have few negative effects on bees.

The FA treatment acts rapidly on varroa in the dispersal and reproduction phases (Bolli et al. 1993), which explains the increased varroa drop in the week following its application. Its acaricidal effect is also maintained in the hive for at least 14 days (Giovenazzo and Dubreuil 2011). The OA treatment works differently as it acts by contact with mites during their dispersal phase (Gregorc and Planinc 2002, Rademacher and Harz 2006, Qadir et al. 2021). In this study, its acaricidal effect was not observed during the 3 weeks following application (no difference of varroa drop from the control group). However, during the application of the Apivar, there was significantly higher varroa drop in the control group colonies indicating that there were less residual varroa mites after the OA summer treatment. Unfortunately, this reduced varroa load was not sufficient to significantly reduce mortality. The mode of action of OA/glycerin pads appears to be slow, and this delayed action may not have resulted in sufficient efficacy to reduce the impact on winter bees (Martin et al. 2010) when applied only once at the beginning of August. This could explain why colony mortality was not reduced.

FA is a volatile compound, and our analyses show that it accumulates in honey following treatment, as reported in the literature (Hansen and Guldborg 1988, Bogdanov et al. 2002). However, since FA is naturally present in honey, there is no maximum residue limit. Nevertheless, Bogdanov et al. (2002) suggest that the taste detection threshold, which is around 150–600 mg/kg, should not be exceeded (Bogdanov et al. 1999). The amount of FA in honey is variable and depends on the floral species from which it is derived. For example, *Castanea sativa* honey can contain more than 500 mg/kg of FA (Suárez-Luque et al. 2006). Formic Pro treatment increased significantly FA concentration in honey samples compared to untreated control group honey (mean = 336 and 39.5 mg/kg, respectively) exceeding the taste detection threshold. However, the honey samples were taken directly from the super closest to the treatment, which could overestimate the amount of FA residue. Furthermore, FA evaporates over time until it reaches the amount naturally found in honey from an untreated hive. This evaporation can continue for up to 8 months (Stoya et al. 1986).

OA and glycerin are naturally present in honey and so were detected in the honey of the control and OA group. A greater amount of OA was detected in the control group, but this amount was not significantly different from the amount measured in the OA group. Similar amounts of glycerin were detected in the control and OA group. Thus OA/glycerin pads, used as described in this project, did not leave residues in honey.

### Viruses and Mortality

The application of a summer treatment had no significant effect on the prevalence of the 6 tested viruses (ABPV, BQCV, DWV-A, DWV-B, IAPV, and KBV) and on the colonies’ viral load except for BQCV. Although the FA treatment significantly reduced the varroa population during the production of winter bees, these bees were not significantly less infected, and on the contrary, the FA group was even more infected by BQCV in April. The reduction of mortality observed in the FA group cannot be linked to a viral load reduction of these viruses in this study. In this case, maybe the reduction of mortality could rather be explained by a reduction in physical injuries caused by varroa and their consumption of honey bee hemolymph and body fat (Ramsey et al. 2019). Such physical damages are known to affect the development of physiological characteristics that characterize winter bees and ensure their increased longevity such as body fat reserves (Kovac and Crailsheim 1988, Amdam et al. 2004). Several studies demonstrated that varroa is a vector of several viruses (Allen et al. 1986, Shen et al. 2005, Berthoud et al. 2010, Di Prisco et al. 2011, Borba et al. 2022) and allows the replication of some, such as ABPV and DWV (de Miranda and Genersch 2010, Gisder et al. 2018). Data of this project demonstrate that a varroa drop rate below the fall IPM threshold of 15 daily varroa drop at the end of the summer did not reduce the prevalence and viral load of the 6 tested viruses at the colony level, while reducing colony mortality. Our results lead us to believe that the physical damages caused by varroa feeding activity may be as harmful at the colony level as the presence of viruses and may contribute to the synergy between viruses and varroa mites (Kuster et al. 2014). However, it remains clear that colony mortality as well as the DWV-B are a problem in Canada, as reported in other countries (Kevill et al. 2019, Martin and Brettell 2019, Paxton et al. 2022), and they may be correlated (Natsopoulou et al. 2017).

Most viruses whose presence was documented in this study (ABPV, DWV-A, DWV-B, and KBV) have a higher prevalence in April, probably because of the long winter confinement that creates an environment conducive to the transmission of these viruses. A higher viral load was also measured in April for ABPV, DWV-A, and DWV-B, and we found no IAPV in April. For BQCV, a higher viral load was measured in July.

The present study shows the potential of adding a summer treatment in an IPM of *V. destructor*. Using this strategy, beekeepers could reduce colony mortality, but improvements and further studies need to be conducted before a new slow-release OA treatment can be marketed in Canada.

## Supplementary Material

ieae042_suppl_Supplementary_Tables_1_Figures_1-2
