# Supplementary material for: Effects of summer treatments against Varroa destructor on viral load and colony performance of Apis mellifera colonies in Eastern Canada
Source: J Insect Sci. 2024 May 28;24(3):14. doi: 10.1093/jisesa/ieae042 (PMC11132135; doi:10.1093/jisesa/ieae042)
Supplement: ieae042_suppl_Supplementary_Tables_1_Figures_1-2 [file ieae042_suppl_supplementary_tables_1_figures_1-2.zip › Supplementary files Fig and Table/Supplementary files.docx]

**Supplementary Table 1.** Primers and probes used for virus analysis.

|  | | Primers (5’ - > 3’) | Probe (5’ - > 3’) | Reference | Genbank Acess No. |
| --- | --- | --- | --- | --- | --- |
| Virus | | | | | |
| Multiplex 1 | ABPV | F: CATATTGGCGAGCCACTATG  R: CTACCAGGTTCAAAGAAAATTTC | ATAGTTAAAACAGCTTTTCACACTGG | Ciglenečki et Toplak 2012 | AF486072.2 |
|  | DWV A | F: TTCATTAAAGCCACCTGGAACATC  R: TTTCCTCATTAACTGTGTCGTTGA | TGTTATCTCCTGCGTGGAATGCGT* | Locke et al. 2012  *this study | MT415949.1 |
|  | DWV B | F: TATCTTCATTAAAACCGCCAGGCT  R: CTTCCTCATTAACTGAGTTGTTGTC | TGAGAGGGATGAGACCTGAACTTG* | McMahon et al. 2015  *this study | MT415952.1 |
| Multiplex 2 | BQCV | F: GGTGCGGGAGATGATATGGA  R: GCCGTCTGAGATGCATGAATAC | TTTCCATCTTTATCGGTACGCCGCC | Chantawannakul et al. 2006 | MT482476.1 |
|  | IAPV | F: GCGGAGAATATAAGGCTCAG  R: CTTGCAAGATAAGAAAGGGGG | CGCCTGCACTGTCGACATTAGTTA* | Di Prisco et al. 2011  *this study | KY243933.1 |
|  | KBV | F: ACCAGGAAGTATTCCCATGGTAAG  R: TGGAGCTATGGTTCCGTTCAG | CCGCAGATAACTTAGGACCAGATCAATCACA | Ward et al. 2007 | AF263723.1 |
| Reference gene | | | | | |
| Multiplex 1 and 2 | $\beta$-actin | F: AGGAATGGAAGC TTGCGGTA  R: AATTTTCATGGTG GATGGTGC | ATGCCAACACTGTCCTTTCTGGAGGTA | Chen et al. 2005 | AB023025.1 |


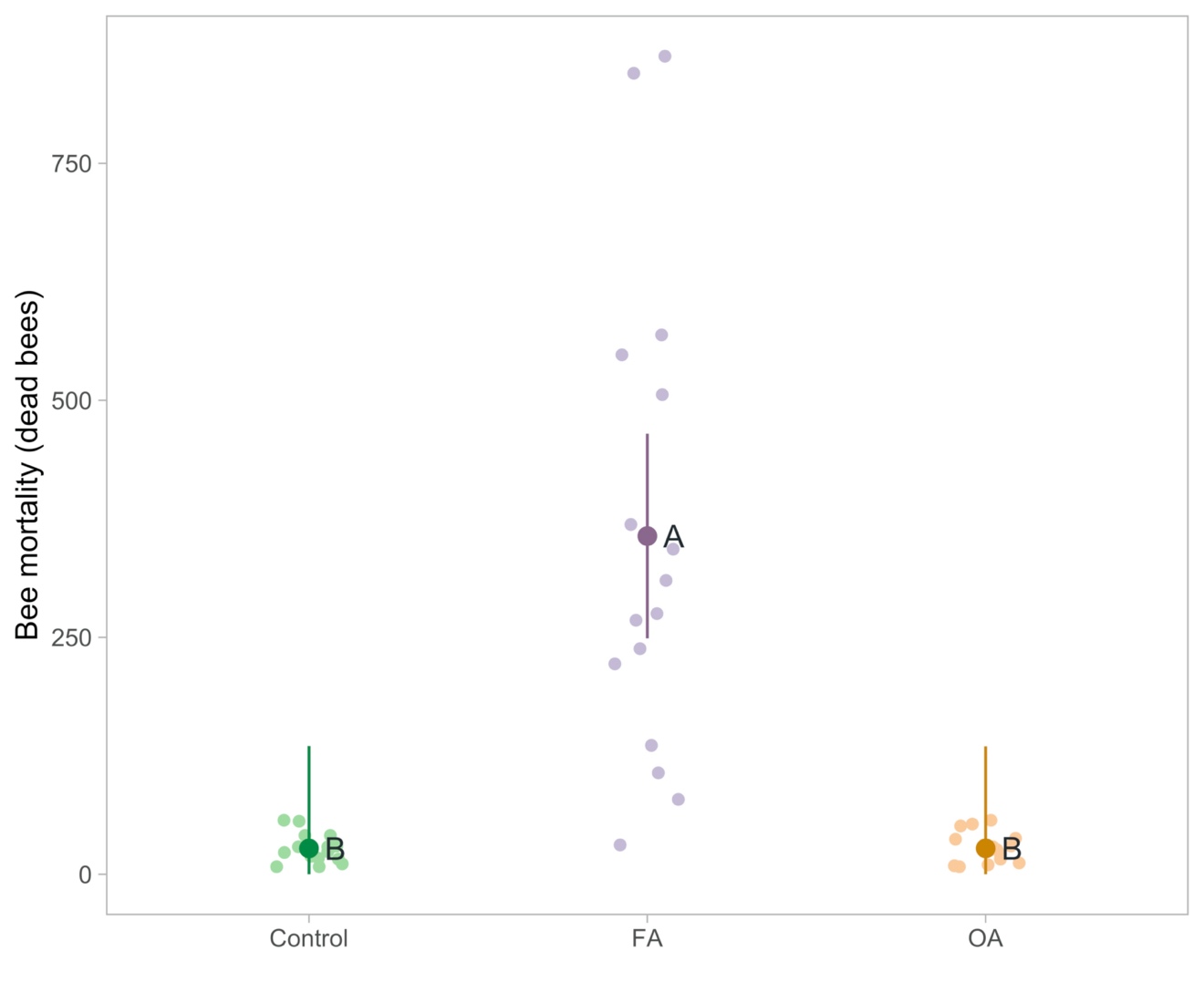


**Supplementary Fig. 1.** Honey bee mortality (dead bees) in control group (n = 16), formic acid (FA) group (n = 16) and oxalic acid (OA) group (n = 15). Measured from 2 to 7 August 2021. The mean and 95% confidence interval were estimated by the model with untransformed data and each point represents the observed value of a single colony. A statistical difference between groups is indicated by different letters next to the estimate (*p* ≤ 0.0001).

**
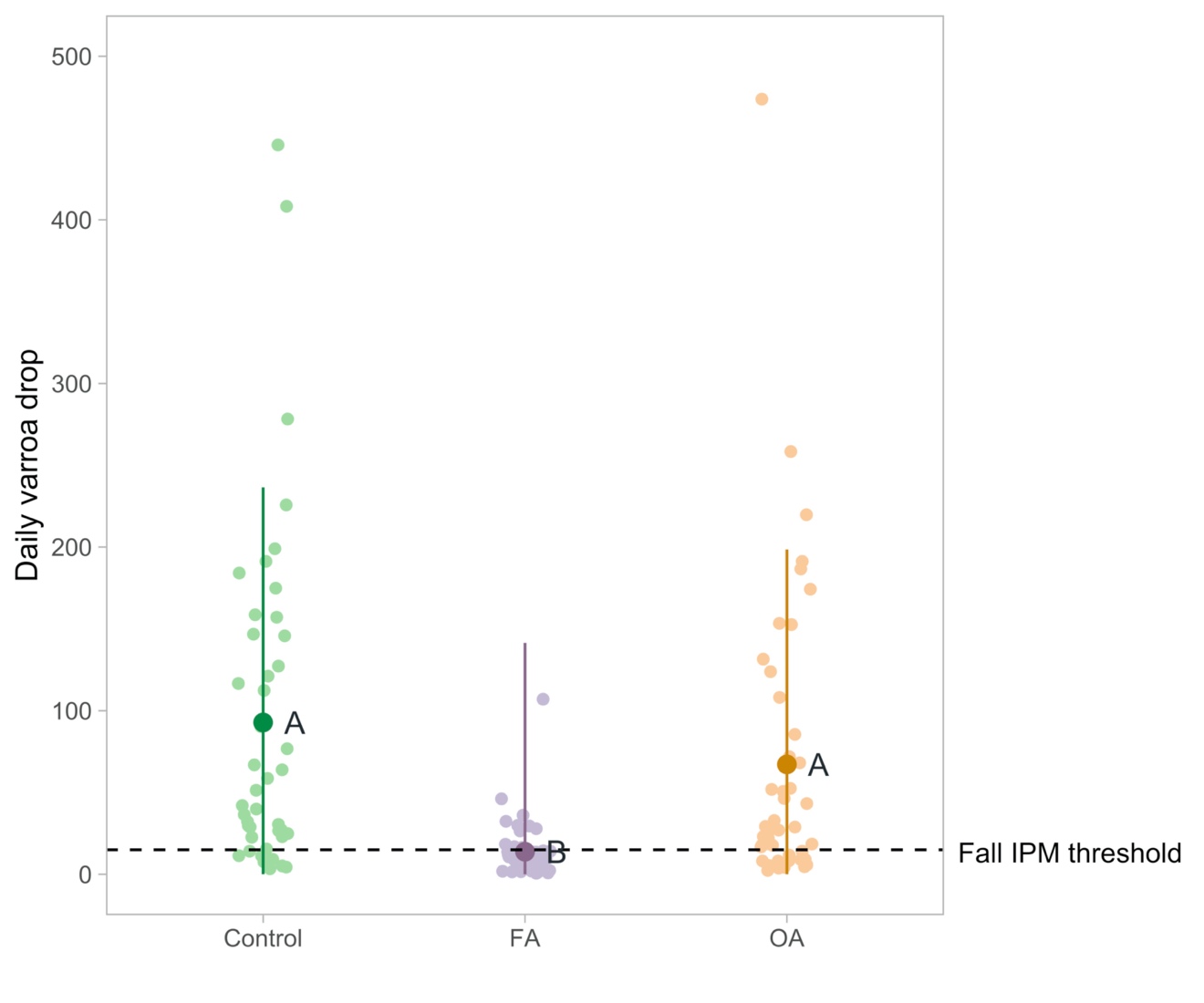
Supplementary Fig. 2.** Daily varroa drop at the end of the summer in the control group (*n*= 43), formic acid (FA) group (*n* = 45) and oxalic acid (OA) group (*n*= 45). Measured on 30 August 2021. The mean and 95% confidence interval were estimated by the model with untransformed data and each point represents the observed value of a single colony. A statistical difference between groups is indicated by different letters next to the estimate (*p* ≤ 0.0001).

## References

**Chantawannakul, P., L. Ward, N. Boonham, and M. Brown. 2006.** A scientific note on the detection of honeybee viruses using real-time PCR (TaqMan) in Varroa mites collected from a Thai honeybee (*Apis mellifera*) apiary. J. Invertebr. Pathol. 91(1): 69–73. <http://doi.org/10.1016/j.jip.2005.11.001>.

**Chen, Y. P., J. A. Higgins, and M. F. Feldlaufer. 2005.** Quantitative real-time reverse transcription-PCR analysis of deformed wing virus infection in the honeybee (*Apis mellifera* L.). Appl. Environ. Microbiol. 71(1): 436–441. <http://doi.org/10.1128/AEM.71.1.436-441.2005>.

**Ciglenečki, U. J. and I. Toplak. 2012.** Development of a real-time RT-PCR assay with TaqMan probe for specific detection of acute bee paralysis virus. J. Virol. Methods. 184(1-2): 63–68. <http://doi.org/10.1016/j.jviromet.2012.05.010>.

**Di Prisco, G., F. Pennacchio, E. Caprio, H. F. Boncristiani, J. D. Evans, and Y. Chen**. **2011**. *Varroa destructor* is an effective vector of Israeli acute paralysis virus in the honeybee, *Apis mellifera*. J. Gen. Virol. 92(1): 151–155. <http://doi.org/10.1099/vir.0.023853-0>.

**Locke, B., E. Forsgren, I. Fries, and J. R. de Miranda. 2012.** Acaricide treatment affects viral dynamics in *Varroa destructor*-infested honey bee colonies via both host physiology and mite control. Appl. Environ. Microbiol. 78(1): 227–235. <http://doi.org/10.1128/AEM.06094-11>.

**McMahon, D. P., M.A. Fürst, J. Caspar, P. Theodorou, M. J. Brown, and R. J. Paxton. 2015.** A sting in the spit: widespread cross‐infection of multiple RNA viruses across wild and managed bees. J. Anim. Ecol. 84(3): 615–624. <http://doi.org/10.1111/1365-2656.12345>.

**Ward, L., R. Waite, N. Boonham, T. Fisher, K. Pescod, H. Thompson, P. Chantawannakul, and M. Brown. 2007.** First detection of Kashmir bee virus in the UK using real-time PCR. Apidologie 38(2): 181–190. <http://doi.org/10.1051/apido:2006072>.
