# Supplementary figures and images for: Effects of summer treatments against Varroa destructor on viral load and colony performance of Apis mellifera colonies in Eastern Canada
Source: J Insect Sci. 2024 May 28;24(3):14. doi: 10.1093/jisesa/ieae042 (PMC11132135; doi:10.1093/jisesa/ieae042)

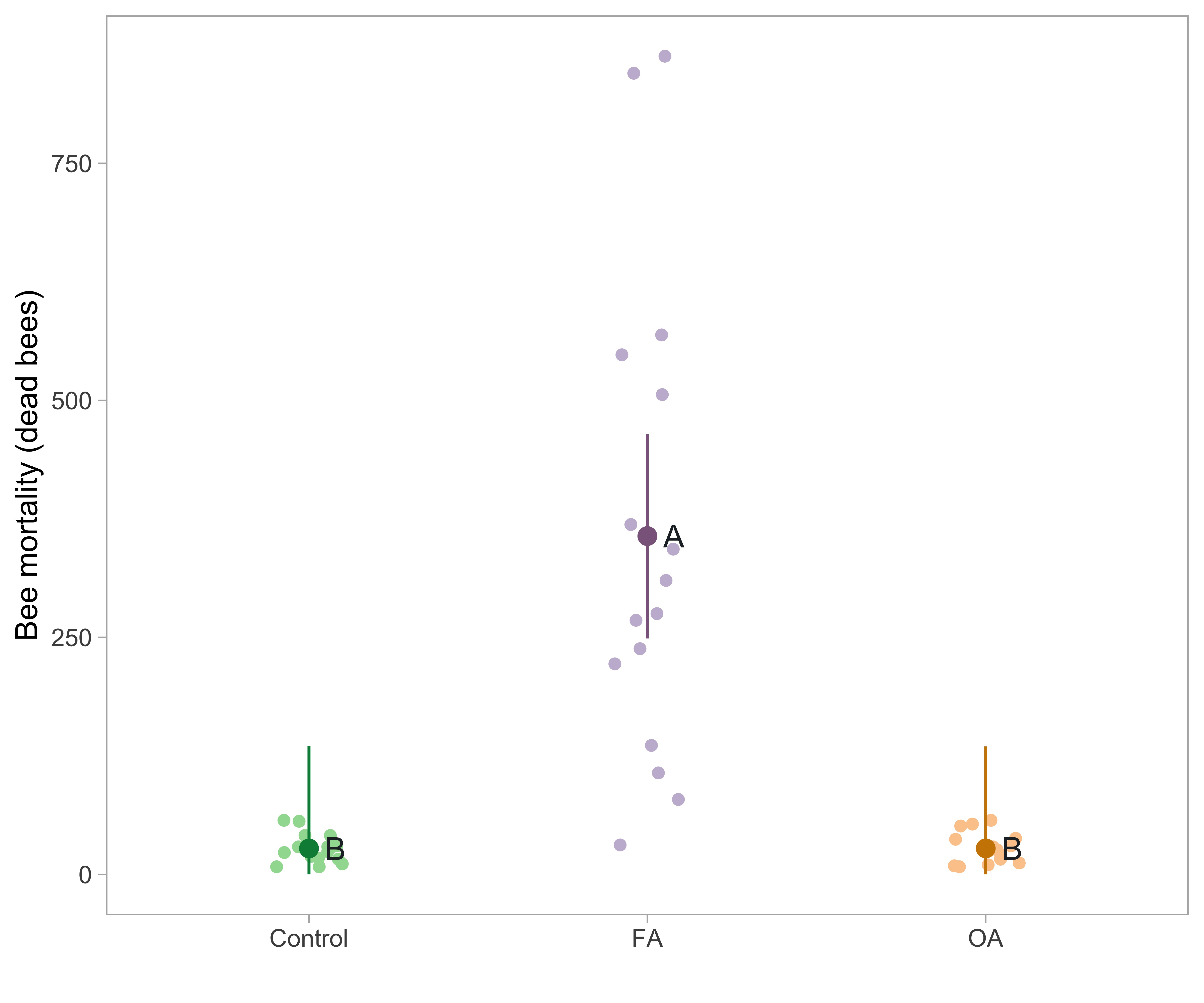

Supplement: ieae042_suppl_Supplementary_Tables_1_Figures_1-2 [file ieae042_suppl_supplementary_tables_1_figures_1-2.zip › Supplementary files Fig and Table/Supplementary Fig. 1.tiff]

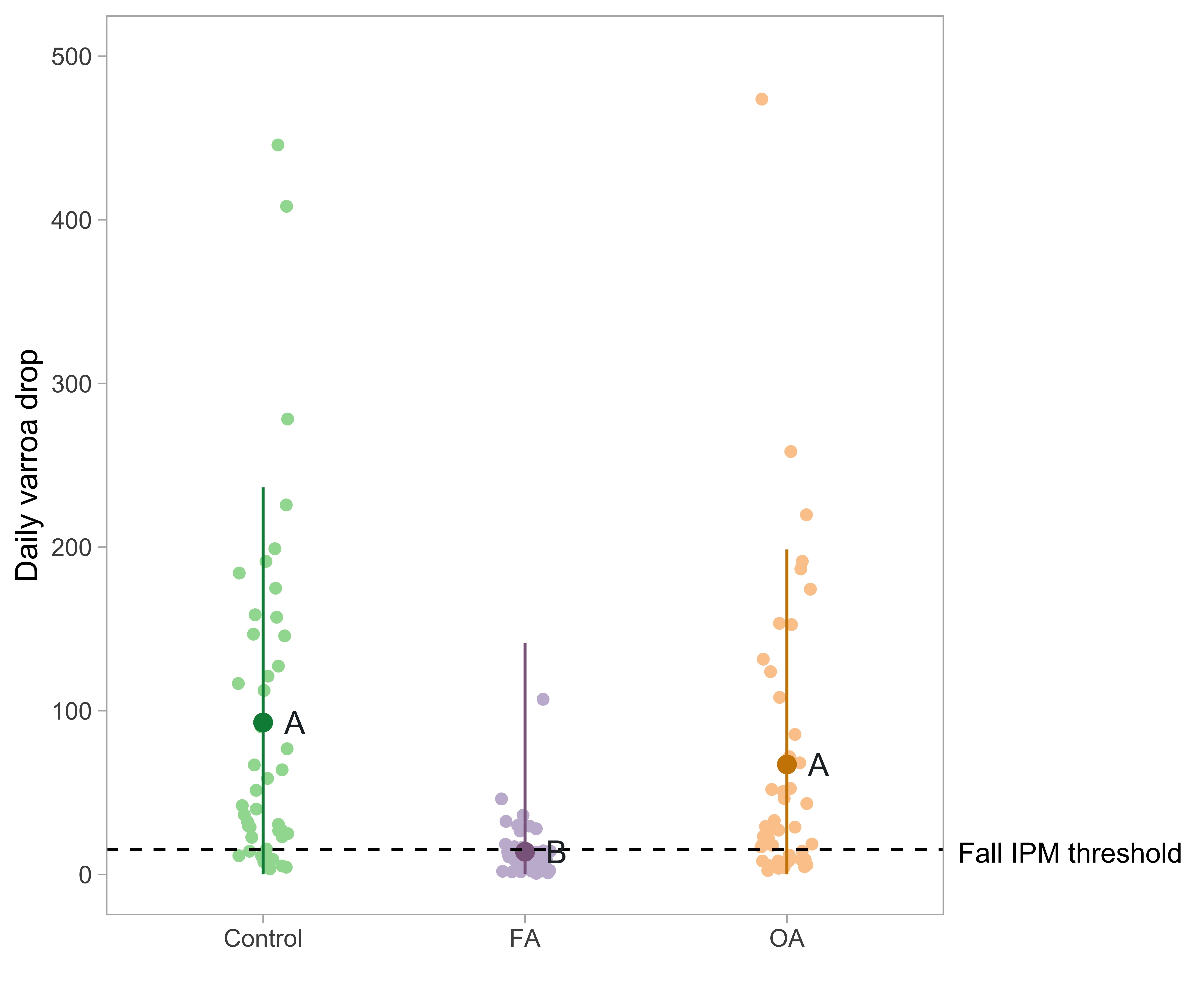

Supplement: ieae042_suppl_Supplementary_Tables_1_Figures_1-2 [file ieae042_suppl_supplementary_tables_1_figures_1-2.zip › Supplementary files Fig and Table/Supplementary Fig. 2.tiff]
